# Are We Asking All the Right Questions About Quality of Care in Low- and Middle-Income Countries?

**DOI:** 10.15171/ijhpm.2018.48

**Published:** 2018-05-27

**Authors:** Stephanie M. Topp, Kabir Sheikh

**Affiliations:** ^1^College of Public Health, Medical and Veterinary Sciences, James Cook University, Townsville, QLD, Australia.; ^2^Public Health Foundation of India, New Delhi, India.; ^3^Nossal Institute for Global Health, University of Melbourne, Melbourne, VIC, Australia.

## Dear Editor,

Quality of care (QoC) – what it is and how to achieve it – is a hot topic in Global Health. Contextualised by the widespread interest in universal health coverage (UHC) reforms in low- and middle-income countries (LMICs) a number of high-profile initiatives and networks now exist on the topic (eg, *The Lancet Global health Commission on High Quality Health*, the *Network for Improving Quality of Care for Maternal, newborn and Child Health*, *The Primary Health Care Performance Initiative*).^1-3^ These initiatives reflect growing recognition of the need for high quality and safe care in reducing persistent differences in global health outcomes. Yet there are opportunities for the global health agenda for QoC to be better informed by the characteristics of the health systems through which it would be realised.^4,5^ The global movement for QoC must find its moorings in the complex realities of LMIC health systems, in order to be effective in catalysing improvements on the ground. To this end we have the following suggestions for QoC advocates and researchers:

### Disrupt Simple Public vs. Private Dichotomies – They Don’t Reflect the Reality of Health Systems

Much of the global debate on QoC focuses on drawing comparisons between the public and private health sectors.^6,7^ Seeking to artificially separate and compare QoC across these sectors neglects their heavily overlapping organisational, social and economic context and shared history, and propagates a false message that policy choices in regard to the public private mix are binary. Privately motivated behaviour (either planned through specific schemes to introduce market logic and incentives, or unplanned) abounds in public sector healthcare delivery. In most LMIC health systems, there is a characteristic blurring of the public and private sector, and the public private mix is therefore more helpfully conceptualised as a spectrum than a dichotomy.^8-10^ What does bear more detailed investigation is the nature of these overlaps, and their influence on the experiences of service users. For instance, we do not know well enough (from LMIC contexts) what the impact is of introducing different market models – partnerships and incentives – on the quality of public sector services. Or of how variable state capacity to regulate and purchase services strategically influences the quality of private healthcare.

### Look Beyond Health Worker Performance – Structural Factors Determine QoC

A distinct, but related trend in the literature is frequent conflation of the concepts of QoC, and health worker performance.^11^ To be sure, users most often experience the health system through health workers, and as such, health worker performance both in relation to technical capability and person-centeredness are critical. Yet health worker performance is only one component of QoC. Conflation of the two concepts tends to (unfairly) place implicit responsibility for QoC on frontline health workers in LMICs. The conflation of quality and performance also diverts attention from equally important and pervasive structural influences on QoC such as market and governance failures,^9^ ‘practical norms’ that apply across the system,^12^ and workplace and patient provider trust and respect.^13-15^ In doing so, it can promote short sighted policies that target health workers alone (eg, stand-alone performance based financing or training interventions) while reforms targeting broader structural determinants of those problems are overlooked.

### Ask How QoC Can Be Improved, and Who Can Improve it?

Considering that healthcare is provided in such varied social and organizational contexts, there is currently a bias towards standardisation and international comparability in global research on QoC, putatively addressed to a global audience of decision makers.^16,17^ Research on QoC is likely to be more effective if it explicitly considers how it will lead to improvements *in context*, and engages the full range of people and institutions capable of bring about the desired improvements. A broader palette of methodological approaches than is currently in use is thus warranted, to respond to complex and varied health system contexts. Qualitative social science and “embedded” approaches in implementation science can help understand the social, organizational and relational determinants of QoC, and need to be applied to complement the more quantitative forms of enquiry and evaluation that are currently privileged.^4^ Global research on QoC has made strides in embracing the perspectives of service users.^18,19^ However it also needs to include decision-makers at national and sub-national levels – planners, regulators, managers and healthcare providers – as co-producers of research and directly address their knowledge needs. Those closer to the desired changes are best equipped to make them happen.

## Ethical issues

Not applicable.

## Competing interests

Authors declare that they have no competing interests.

## Authors’ contributions

Both authors conceived of the article. ST wrote the first draft. Both authors shared equally in the editing and refining of the manuscript.

## Authors’ affiliations

^1^College of Public Health, Medical and Veterinary Sciences, James Cook University, Townsville, QLD, Australia. ^2^Public Health Foundation of India, New Delhi, India. ^3^Nossal Institute for Global Health, University of Melbourne, Melbourne, VIC, Australia.
